# Bloodmeal metabarcoding reveals host feeding patterns for Aedes aegypti and Culex quinquefasciatus in Jutiapa, Guatemala and Texas, USA

**DOI:** 10.21203/rs.3.rs-8855613/v1

**Published:** 2026-02-19

**Authors:** Abdisalam A. Abdi, Sujata Balasubramanian, Jose Juarez, Nicole A. Scavo, Nadia A. Fernandez-Santos, Yuexun Tian, Sarah A. Hamer, Pamela Pennington, Norma Padilla, Gabriel L. Hamer

**Affiliations:** Texas A&M University; Texas A&M University; Texas A&M University; Texas A&M University; Texas A&M University; Texas A&M University; Texas A&M University; Universidad del Valle de Guatemala; Universidad del Valle de Guatemala; Texas A&M University

**Keywords:** bloodmeal analysis, metabarcoding, Aedes aegypti, Culex quinquefasciatus, forage ratio, arbovirus transmission

## Abstract

Mosquito host contact determines arboviral transmission efficiency. *Aedes aegypti* and *Culex quinquefasciatus* are important vectors of dengue, Zika, chikungunya, West Nile virus, and other arboviruses, yet their feeding patterns remain poorly characterized in many tropical regions. We used bloodmeal metabarcoding to detect DNA from multiple vertebrate species within individual blood-fed mosquitoes collected from rural Guatemala and south Texas, USA. Mosquitoes were collected using aspiration in Guatemala and BG-Sentinel traps in south Texas. We calculated forage ratios (FR) to assess host utilization relative to availability. In Guatemala, *Ae. aegypti* exhibited strong anthropophilic behavior (human DNA: 90.2% of bloodmeals and FR = 3.62 (95% CI: 2.70–4.54), indicating significant over-utilization. In south Texas, *Ae. aegypti* strongly over-utilized dogs (88.2% of bloodmeals; FR = 4.65, 95% CI: 2.43–6.87) while under-utilizing humans (FR = 0.53, 95% CI: 0.25–0.81). In Guatemala, *Cx. quinquefasciatus* displayed high anthropophilic behavior (85.3% of bloodmeals; FR = 2.60, 95% CI: 2.24–2.97). Mixed bloodmeals were common in both species at both sites (19.5–85.3%), with up to four host species detected in single mosquitoes. These results demonstrate that mosquito host selection is variable and context-dependent and underscore the need for location-specific surveillance to inform vector control strategies.

## Introduction

Mosquito-borne pathogens cause over 700,000 deaths annually and impose billions of dollars in economic costs worldwide [1]. These pathogens include malaria (*Plasmodium spp*.), dengue virus (DENV), Zika virus (ZIKV), chikungunya virus (CHIKV), yellow fever virus (YFV), West Nile virus (WNV), and Japanese encephalitis virus (JEV) [2, 3]. These diverse pathogens exist in cycles between competent mosquito vector species and competent amplification vertebrate host species [4, 5]. In some cases, humans function as amplification hosts and develop an infectious viremia capable of reinfecting a mosquito [6, 7]. When humans function as amplification hosts and transmission is maintained primarily between mosquitoes and humans in built environments, this pattern is known as an “urban cycle” or a “human-amplified transmission cycle”. This transmission pattern characterizes several major pathogens transmitted by *Aedes aegypti*, including DENV, ZIKV, and CHIKV [8–10]. Alternatively, other pathogens such as WNV and eastern equine encephalitis virus (EEEV) utilize wild or domestic animals as amplification hosts which are fed on by diverse mosquito species and maintained in what are known as ‘sylvatic cycles’ [11, 12]. As these mosquito-borne pathogens amplify in the mosquito and animal cycle, a bridge vector species is capable of spill-over transmission to humans or other domestic animals which can suffer disease, while not necessarily being capable of developing an infectious viremia (i.e., dead-end host for virus) [5, 6, 13].

The type of transmission cycle is fundamentally linked to the host-feeding behavior of vector mosquito species [14–16]. Anthropophilic species such as *Ae. aegypti* preferentially feed on humans [17–20], which makes this species a potent vector of human pathogens in tropical and subtropical regions. In contrast, ornithophilic (bird-feeding) species like members of the *Culex pipiens* complex maintain sylvatic and zoonotic cycles by feeding primarily on avian hosts [21–23]. However, some studies have documented atypical non-human feeding by *Ae. aegypti* in both South Texas [24], and Kenya [25], which may reduce mosquito–human contact rates and thereby lower transmission potential for human-amplified arboviruses. Therefore, variation in *Ae. aegypti* host use can shape the intensity and spatial pattern of *Aedes*-borne pathogen transmission [15, 26, 27].

An important mosquito vector of arthropod-borne viruses (arboviruses) globally are members of the *Culex pipiens* complex. This complex includes the northern house mosquito, *Cx. pipiens pipiens*, in more temperate latitudes globally, and the southern house mosquito, *Culex. quinquefasciatus*, found in more tropical and subtropical regions globally [28]. The feeding patterns of species belonging to the *Cx. pipiens* complex has been extensively studied [29, 30], given that they are important in the transmission of many zoonotic pathogens. However, most of this research attention on *Culex* spp. mosquitoes has been in temperate regions while vectors in this genus have been neglected in many tropical and subtropical regions which are endemic for human-amplified mosquito-borne pathogens such as malaria and dengue [31]. Many studies report an ornithophilic feeding pattern of *Cx. pipiens* complex [21–23, 32–34]. However, *Cx. quinquefasciatus* feeding patterns are more complex with some studies reporting high utilization of birds as hosts while others document high feeding rates on mammals, including humans [35, 36].

This uncertainty in the feeding patterns of *Cx. quinquefasciatus* may also be driven by few bloodmeal analysis studies being conducted in tropical and subtropical regions. Recent meta-analyses reveal that *Cx. quinquefasciatus* feeding patterns vary substantially across biogeographic regions, with some tropical populations exhibiting predominantly mammalian or human feeding rather than avian feeding [37]. Beyond this geographic variability, accurately characterizing mosquito host use is further complicated by the feeding behavior of key vector species. Both *Ae. aegypti* and *Culex* spp. are known to take multiple bloodmeals from different host species within a single gonotrophic cycle, increasing the frequency of mixed-species bloodmeals [15, 38]. Traditional PCR followed by Sanger sequencing has been the primary method for bloodmeal analysis, but it has critical limitations: it typically detects only the most abundant DNA in a sample, often missing hosts in mixed bloodmeals [39, 40]. For example, our previous study in South Texas used PCR-Sanger sequencing documented *Ae. aegypti* feeding patterns [41], but this approach may have underestimated the frequency of human feeding in mixed bloodmeals where dog DNA was preferentially amplified in the PCR. These methodological constraints mean that our understanding of mosquito host-feeding patterns may be incomplete, particularly for species that frequently feed on multiple hosts. High-throughput sequencing methods can overcome these limitations by detecting most vertebrate DNA present in a bloodmeal, regardless of abundance. Specifically, bloodmeal metabarcoding employs deep sequencing of vertebrate-specific DNA loci, enabling detection of multiple host species within bloodmeals from individual arthropods [42, 43]. This approach has been successfully applied to characterize host-feeding patterns in various hematophagous arthropods, including mosquitoes [44, 45], soft ticks [43], and triatomines [46].

Given the uncertainty of *Ae. aegypti* and *Cx. quinquefasciatus* feeding patterns in tropical regions, where most previous bloodmeal studies employed Sanger sequencing with limited capacity to detect mixed-species meals, the objective of this study was to conduct bloodmeal metabarcoding on field-collected females of both *Ae. aegypti* and *Cx. quinquefasciatus* species from rural communities in Guatemala and south Texas, USA. The work in south Texas builds on our previous work using PCR-Sanger sequencing, which documented high use of non-human animals [24]. We will now verify these unexpected feeding patterns using new mosquito collections from different years, different study sites, and using the bloodmeal metabarcoding pipeline. The collections in Guatemala provide an opportunity to utilize this novel metabarcoding pipeline in a tropical setting. This study provides a unique opportunity to study multiple bloodfeeding behaviors within the same gonotropic cycle, which is well-documented for *Ae. aegypti* [15, 17, 47–50] but less understood for *Culex* spp. mosquitoes [51, 52].

## Results

### Guatemala

A total of 243 blood-fed female mosquitoes were collected from 77 households during the 2022 rainy season in Guatemala. Of these, 228 (93.8%) were molecularly confirmed to species: 67 *Ae. aegypti* (29.4%), 2 *Ae. albopictus* (0.9%), 155 *Cx. quinquefasciatus* (68.0%), 2 *Cx. nigripalpus* (0.9%), and 2 *Cx. corniger* (0.9%). All molecular identifications matched morphological assignments.

Of the 67 *Ae. aegypti* tested, 41 (61.2%) produced PCR product and sequencing reads that yielded host identifications. Among the specimens with results, 33 (80.5%) were single-host meals, including 29 human (*Homo sapiens*) and 4 chicken (*Gallus gallus*). The remaining 8 (19.5%) meals were mixed, consisting of 6 human-chicken combinations and 2 human-bird (*Turdus* sp.) combinations. Humans were detected in 37/41 (90.2%) of the *Ae. aegypti* bloodmeals, chickens in 10/41 (24.4%), and *Turdus* sp. in 2/41 (4.9%) (Table 1). Forage-ratio inference indicated over-utilization of humans relative to availability (FR = 3.62, 95% CI 2.70–4.54), whereas feeding on chickens was not significantly different from availability (FR = 1.25, 95% CI 0.41–2.08) (Table 2). Both *Ae. albopictus* (n = 2) yielded identifiable meals: one single-host (human) and one mixed-host (human-chicken).

Among blood-fed *Cx. quinquefasciatus* processed (n = 155), 136 (87.7%) yielded host identifications. Of these, 48 were single-host meals (35.3%), including 31 human, 15 chicken, 1 turkey (*Meleagris gallopavo*), and 1 passerine bird (*Passeriformes*). A further 70 (51.5%) were mixed two-host meals, composed of 61 human–chicken, 5 human–dog (*Canis lupus familiaris*), 1 human–turkey, 2 chicken–dog, and 1 chicken–turkey. The remaining 18 (13.2%) were mixed three-host meals, including 16 human–chicken–dog, 1 human–chicken–turkey, and 1 human–chicken–bird (*Turdus sp*.) (Table 1). For the less common mosquito species, both *Cx. nigripalpus* (n = 2) contained single-host meals 1 human and 1 chicken, while the single *Cx. corniger* (n = 1) specimen yielded a mixed two-host meal (human–chicken). Across all *Cx. quinquefasciatus* bloodmeals, humans were detected in 116/136 (85.3%), chickens in 97/136 (71.3%), and dogs in 21/136 (15.4%) (Table 1). For the *Cx. quinquefasciatus* forage-ratio, humans were significantly over-utilized (FR = 2.60, 95% CI 2.24–2.97), chickens were marginally over-utilized (FR = 1.27, 95% CI 1.07–1.46), and utilization of dogs did not differ from availability (FR = 0.99, 95% CI 0.51–1.47) (Table 2).

### South Texas, USA

Of 121 blood-fed *Ae. aegypti* examined in south Texas, 68 (56.2%) yielded at least one host identification. Of these, 10 (14.7%) were single-host meals, including 2 human (*Homo sapiens*), 3 dog (*Canis lupus familiaris*), 1 chicken (*Gallus gallus*), 3 house mouse (*Mus musculus*), and 1 brown rat (*Rattus norvegicus*). The remaining 58 (85.3%) bloodmeals were mixed, including 35 (51.5%) two-host meals, 19 (27.9%) three-host meals, and 4 (5.9%) four-host meals. These consisted of 25 human–dog, 7 dog–cat (*Felis catus*), 2 chicken–dog, 1 chicken–mouse, 17 human–dog–cat, 1 human–dog–rat, 1 chicken–dog–mouse, and 4 human–dog–cat–rat.

Across all meals, dogs were detected in 60/68 (88.2%) of bloodmeals, humans in 49/68 (72.1%), cats in 28/68 (41.2%), brown rats in 6/68 (8.8%), house mice in 5/68 (7.4%), and chickens in 5/68 (7.4%) (Table 1).

Forage-ratio inference indicated significant under-utilization of humans relative to their abundance (FR 0.53, 95% CI 0.25–0.81) and strong over-utilization of dogs (FR 4.65, 95% CI 2.43–6.87). Cats did not differ from availability (FR 1.55, 95% CI 0.52–2.57), and chicken FRs were not calculated because household availability was zero for most records (Table 2).

### Mixed feeding comparisons in Guatemala and Texas

Mixed feeding patterns differed significantly between mosquito species within Guatemala; *Cx. quinquefasciatus* exhibited higher rates of mixed feeding than *Ae. aegypti* (64.7% vs. 19.5%; χ^2^ = 24.13, df = 1, p < 0.001) (Table 1). South Texas *Ae. aeg*ypti showed the highest mixed feeding rate at 85.3%, significantly greater than Guatemala *Ae. aegypti* (χ^2^ = 43.62, df = 1, p < 0.001) (Table 1).

### Household Characteristics

Household surveys documented substantial differences in housing infrastructure between study sites. In Guatemala, none of the 77 households had air conditioning and all lacked intact window or door screens. In South Texas, 46 of 48 surveyed households (95.8%) had air conditioning (19 central systems, 27 window units), and most had screened windows or doors.

## Discussion

Our bloodmeal metabarcoding revealed substantial geographic heterogeneity in the host use of *Ae. aegypti* and *Cx. quinquefasciatus* across south Texas and rural Guatemala, including shifts between human and domestic animal feeding with implications for arboviral epidemiology. In Guatemala, *Ae. aegypti* exhibited anthropophilic feeding behavior, with human DNA being detected in 90.2% of the identified bloodmeals, and the human FR being 3.62 ± 0.45 (2.70–4.54) (Table 2), indicating significant over-utilization of humans relative to their abundance. This high anthropophilic behavior aligns with most studies documenting *Ae. aegypti* feeding primarily on humans in domestic environments where close human-mosquito contact is facilitated by lack of physical barriers such as window screens and air conditioning [53]. In contrast, *Ae. aegypti* from south Texas showed substantially lower human feeding (72.1% of identified meals) while dogs were detected in 88.2% of meals. The FR for the south Texas *Ae. aegypti* feeding on humans was 0.53 (95% CI: 0.25–0.81), indicating significant under-utilization of humans relative to their abundance. The south Texas *Ae. aegypti* FR for dog was 4.65 (95% CI: 2.43–6.87) (Table 2) indicating significant over-utilization of dogs. These findings of high utilization of dogs by south Texas *Ae. aegypti* corroborate our prior study from the same region which documented about 31% human bloodmeals, 50% dog, and 19% other vertebrates [24]. This current south Texas study sampled mosquitoes in different years, different south Texas communities, involved different project personnel, and utilized a different bloodmeal analysis technique. While the current study reports bloodmeal metabarcoding results, the prior study conducted PCR-Sanger sequencing methodology [24]. Note that our prior study, which documented 31% of human bloodmeals by PCR-Sanger sequencing, could have missed human meals within mixed bloodmeals while identifying other taxa; conventional PCR followed by direct Sanger sequencing typically yields a single sequence per sample and tends to under-detect minority hosts in mixed bloodmeals, whereas next-generation metabarcoding approaches recover multiple vertebrate hosts from individual mosquitoes and substantially improve the resolution of mixed-source bloodmeals [54, 55]. Consequently, bloodmeals containing both human and dog DNA, where dog DNA was preferentially amplified in the PCR, would often have been classified as “dog only” by PCR Sanger sequencing. With metabarcoding, these meals would have been more accurately identified as mixed (human + dog) meals, helping to explain the higher percentage of human-positive bloodmeals (72%) observed in the current study.

One limitation of the current study is that the mosquito collection method was different in Guatemala compared to south Texas; *Ae. aegypti* in Guatemala were collected by indoor and outdoor aspiration while in south Texas were collected using BG Sentinel 2 traps placed outdoors. The low-income communities in Guatemala often lacked doors, screens, and sometimes walls, and homeowners are experienced with local ministries of health personnel entering the indoor environment for vector surveillance and indoor residual spraying (IRS) of insecticides. Therefore, our field team, which was accompanied by a government employee from the ministries of health, was allowed access to the homes for indoor aspirating. The low-income communities in south Texas are very different Hispanic communities, and our past studies on community engagement have characterized these challenges [56]. Government employees do not routinely enter homes, IRS campaigns are not typically conducted in the US, and therefore our bloodfed mosquito sampling was limited to the outdoor environment. The different sampling collections would be expected to influence the availability of the different hosts, and thus could have influenced feeding patterns. Forage ratios also depend on household-based host availability estimates, which likely undercount free-ranging hosts such as wild birds that are difficult to enumerate. Because metabarcoding detected some wild-bird feeding, forage ratios, particularly those involving avian hosts should be interpreted cautiously and primarily as relative selection was based on the surveyed domestic/peridomestic host community and not wild host community. The lower utilization of humans by *Ae. aegypti* in south Texas could be due to outdoor mosquitoes not having access to the humans due to the presence of doors, windows, and screens. However, studies have documented variable utilization of humans by *Ae. aegypti* using outdoor collections with BG Sentinel traps; human feeding by *Ae. aegypti* collected by BG Sentinel traps in Mombasa, Kenya was about 35% while in Kisumu, Kenya was about 12% [25]. Future studies should interrogate *Ae. aegypti* feeding patterns with more standardized sampling designs to understand differences in the indoor and outdoor environments.

Despite the differences in sampling methods and locations, our results likely reveal important differences in mosquito-human contact patterns that reflect differences in household infrastructure and human behavior. In Guatemala, homes lacked air conditioning and window screens, allowing mosquitoes constant access to indoor spaces where humans spend time. Additionally, many household activities (e.g. washing clothes, food preparation) occurred outdoors, or in kitchens not enclosed by walls on all sides, further facilitating human-mosquito contact. Indeed, high human-feeding rates have been documented in *Ae. aegypti* collected outdoors where humans are readily accessible, as shown in Kenya [57, 58] and India [59], supporting the importance of host accessibility regardless of collection location. In contrast, south Texas households, despite being lower-income communities, typically had air conditioning and screened windows, creating physical barriers that limit mosquito access to humans indoors. Household activities in the south Texas settings are more likely to occur inside, further reducing human-mosquito contact opportunities.

This difference in accessibility may explain the contrasting forage ratios observed: significant over-utilization of humans in Guatemala (FR = 3.62) versus significant under-utilization in South Texas (FR = 0.53). Importantly, the Texas pattern could reflect reduced mosquito access to humans rather than an intrinsic preference for non-human hosts. When barriers limit human accessibility, *Ae. aegypti* could exhibit opportunistic feeding on available hosts, particularly dogs. These findings challenge the traditional view of *Ae. aegypti* as obligately anthropophilic, demonstrating instead that this species exhibits opportunistic feeding behavior when preferred hosts are less accessible. These patterns have important implications for arbovirus transmission. In settings like Guatemala where human-mosquito contact is unrestricted, high anthropophilic feeding maintains intense dengue transmission [60]. In settings like south Texas where physical barriers reduce human accessibility, transmission potential may be lower despite the presence of competent vectors and susceptible human populations, as mosquitoes divert feeding effort to alternative hosts [41].

Understanding the mechanisms underlying this behavioral variation requires consideration of both genetic and ecological factors. The domestic subspecies *Ae. aegypti aegypti* (Aaa), which predominates in the Americas and urban settings globally, is widely considered strongly anthropophilic, whereas the sylvatic African subspecies *Ae. aegypti formosus* (Aef), found only in sub-Saharan Africa, exhibits more generalist feeding on various vertebrate hosts. McBride et al. [61] identified odorant receptor differences associated with human preference in Aaa. Recent population genetics studies in Kenya have revealed that some geographic variation in arbovirus transmission may reflect subspecies differences; Mulwa et al. [62] found coastal Kenyan *Ae. aegypti* populations (Mombasa) are strongly admixed between Aef and Aaa, with Aaa ancestry highest at the coast, while Anyango et al. [63] confirmed western Kenya populations (Kisumu, Busia) are dominated by Aef ancestry. This genetic structure may partly explain Kenya’s pattern of dengue outbreaks occurring in coastal cities but not interior cities. Aaa-enriched coastal populations exhibit higher anthropophily that facilitates transmission, while Aef-dominated western populations remain more zoophilic despite comparable vector competence [25]. In North America, *Ae. aegypti* populations are overwhelmingly *Ae. aegypti aegypti* (Aaa), so the geographic variation in host feeding observed in the Americas is unlikely to be explained by Aaa–Aef subspecies composition alone.

However, substantial geographic variation in host use is also evident among populations likely representing Aaa, underscoring a strong role for local ecology and host accessibility. Our findings from Guatemala and South Texas demonstrate pronounced differences: Guatemala showed 90% human-positive bloodmeals (FR = 3.62) versus South Texas with 72% human-positive but 88% dog-positive (dog FR = 4.65). Similarly, Agha et al. [25] documented feeding differences among Kenyan urban Aaa populations: coastal Mombasa showed 40% human feeding while inland Kisumu showed only 10% (p = 0.03), with Kisumu mosquitoes feeding heavily on dogs, goats, and cows despite human presence. Kamau *et al*. [58] likewise reported higher human blood indices in coastal compared with inland settings. Together, these findings indicate that while genetic background can shape host preference, local host availability and, especially host accessibility mediated by household infrastructure and human behavior can strongly modulate realized feeding patterns, with downstream consequences for arbovirus transmission risk.

We observed similar patterns of context-dependent feeding behavior in *Cx. quinquefasciatus*. Our results for *Cx. quinquefasciatus* feeding patterns in rural Guatemala confirm high human feeding behavior. While members of the *Culex* pipiens complex, including *Cx. quinquefasciatus*, are widely considered strongly ornithophilic [29], we found substantial human feeding alongside chicken feeding, with humans significantly over-utilized relative to their availability. This contrasts with classical descriptions of members of the *Culex pipiens* complex as predominantly bird-feeding mosquitoes. Recent meta-analyses demonstrate that *Culex* feeding patterns are highly variable and context-dependent across biogeographic realms. Griep *et al*. [37] compiled data from 109 publications, representing 29,990 bloodmeals over 15 years, and found that *Culex* feeding patterns were not significantly explained by mosquito phylogeny alone, indicating that external factors play major roles in determining host utilization. Moreover, their analysis of *Cx. quinquefasciatus* across different biogeographical realms revealed significant regional variation in feeding patterns. Based on 10,969 bloodmeals across 40 publications, they found dramatic regional variation: when aggregated across all realms, only about one-third of bloodmeals were avian (34.3%), whereas nearly half were from non-human mammals (48.0%) and 17.4% were from humans, with reptiles accounting for < 1%. Afrotropical (sub-Saharan Africa) populations fed overwhelmingly on non-human mammals (93.4% non-human mammals, 6.2% humans, 0.4% avian), whereas Indomalayan (South and Southeast Asia) populations were predominantly human feeding (62.0% humans, 32.4% non-human mammals, 5.5% avian). In contrast, Australasian (Australia–Pacific) and Neotropical (Central and South America) populations were largely ornithophilic (90.5% and 66.4% avian, respectively), and Nearctic (North American) populations showed a more mixed pattern with roughly equal avian and mammalian feeding (50.1% avian, 18.7% humans, 30.7% non-human mammals, 0.6% reptiles). These realm-specific summaries indicate that the same nominal species can occupy very different feeding niches in different parts of the world. Consistent with this regional variation, field studies have documented high anthropophilic or mammalophilic feeding by *Cx. quinquefasciatus* in tropical settings: 79.8% human feeding in Mauritania [64], and predominantly bovine (57.6%) and human (24.2%) feeding with minimal chicken use (4.2%) in coastal Kenya [65], and mammalophilic populations in Mexico [66]. Together with our Guatemalan findings, these studies demonstrate that *Cx. quinquefasciatus* host use is highly context-dependent. Similar to patterns observed for *Ae. aegypti*, household features and host accessibility likely play important roles in determining *Cx. quinquefasciatus* feeding patterns, as mosquitoes feed opportunistically on available and accessible hosts within their local environment.

Beyond species-specific feeding patterns, our metabarcoding approach revealed important insights into mixed-species feeding behavior. Metabarcoding revealed a high frequency of mixed blood meals. In south Texas, 85.3% of *Ae*. *aegypti* bloodmeals contained DNA from two or more host vertebrate species, while in Guatemala, 19.5% of *Ae. aegypti* had mixed feeding and 64.7% of *Cx. quinquefasciatus* had mixed feeding. While multiple feeding (including partial or interrupted feeding) has been documented in some *Culex* species (e.g., *Cx. tarsalis, Cx. tritaeniorhynchus*) [51, 52], the extent to which members of the *Cx. pipiens* complex routinely take multiple partial bloodmeals, particularly from different host species, remains less well characterized. Mixed-source bloodmeals have been reported in members of the *Cx. pipiens* complex, including *Cx. quinquefasciatus*, although reported frequencies are often low and likely method- and context-dependent [67, 68]. The ability to detect multiple host species within a single bloodmeal represents a methodological advance that may challenge previous understanding of mosquito host selection. Earlier techniques for detecting mixed-species feeding include histological examination, serological methods, species-specific PCRs, or PCR cloning, but these are labor-intensive and offer limited resolution [69–75]. As a result, historical feeding studies may have substantially underestimated the frequency of multiple-host feeding, and these methodological limitations should be considered when interpreting earlier data. For example, Scott et al. [53] found that only about 7% of *Ae. aegypti* in rural Thailand had detectable multiple-host-type bloodmeals, all of which included a human host. Ponlawat & Harrington [18] reported that *Ae. aegypti* in Thailand fed almost exclusively on humans, with multiple-host-type bloodmeals being rare, though they documented that individual mosquitoes frequently took multiple meals from the same host species (humans) within a single gonotrophic cycle. Multiple bloodfeeding within the same gonotrophic cycle increases host-vector contact and pathogen transmission potential [76, 77], whether from the same or different host species. Although detection of multiple host taxa is consistent with mixed feeding within a single gonotrophic cycle, we have not yet evaluated whether metabarcoding may detect residual vertebrate DNA from a prior gonotrophic cycle.

While metabarcoding substantially improves detection of mixed-species feeding, it cannot distinguish multiple individuals of the same host species. Forensic tools such as microsatellites and short tandem repeats can detect unique individual profiles and have identified heterogeneous feeding patterns where *Ae. aegypti* bites concentrate on specific demographic groups or in specific geographic locations, both of which could have important epidemiological consequences [76, 78]. Coupling forensic tools with metabarcoding could enable detection of multiple human individual profiles in human-containing bloodmeals, further refining transmission dynamics analysis [78].

Mosquito host selection is more variable and context-dependent than classical paradigms suggest. *Aedes aegypti* populations are not uniformly anthropophilic; some exhibit high rates of non-human feeding that may reduce arbovirus transmission potential. Similarly, *Cx. quinquefasciatus* feeding behavior varies with local conditions and can be highly anthropophilic in tropical regions.

## Methods

### Study Design

**Figure 1:**
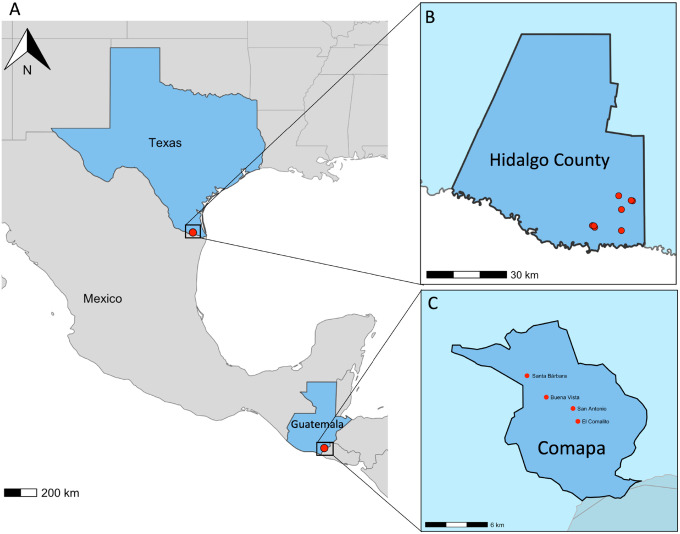
Study regions in South Texas, USA, and Comapa, Guatemala. (A) Regional map showing the southern United States, Mexico, Guatemala, and Nicaragua, with Texas and Guatemala highlighted and points indicating the South Texas (Hidalgo County) and Comapa sampling areas. (B) Inset map of South Texas showing Hidalgo County and study site locations (red points). (C) Inset map of Jutiapa Department showing Comapa municipality and study site locations (red points). Maps were generated in R (sf, ggplot2, ggspatial) using boundaries from Natural Earth (via rnaturalearth), U.S. Census TIGER/Line (via tigris), and GADM (via geodata). Natural Earth data are public domain; TIGER/Line and GADM data were used under their respective terms of use.

We conducted field collections of *Ae. aegypti* and *Cx. quinquefasciatus* from rural Guatemala and south Texas, USA (Fig. 1) and identified blood-fed females to characterize natural host-feeding patterns using bloodmeal metabarcoding. Household surveys were conducted at participating homes in both sites to characterize housing features that may influence mosquito access to indoor environments (e.g., presence of air conditioning and window/door screens) and to support comparisons between Guatemala and south Texas.

The household surveys in Guatemala were reviewed by the Research Ethics Committee of Centro de Estudios en Salud at Universidad del Valle de Guatemala (UVG) which classified it as “Research not involving human subjects” (Protocol No. 270-05-2022). The household surveys in south Texas received approval from the Institutional Review Board of Texas A&M University (IRB2021-0886D). We obtained individual written informed consent from each household owner for the questionnaire. All research was performed in accordance with relevant guidelines and regulations.

## Field collections and sample processing

### Field Sites – Guatemala

Mosquitoes were collected from four communities in the Municipality of Comapa, Department of Jutiapa, Guatemala (Fig. 1). The neighborhoods are in a semi-rural area characterized by poor infrastructure. These communities have had extensive community engagement with investigators from Universidad del Valle de Guatemala during past research projects in partnership with the Ministry of Health [46, 79, 80].

Sampling was carried out during the rainy season (June - August 2022) in 77 households. Prokopack aspirators were used for mosquito collections [81]. All indoor and outdoor sampling with the Prokopack was done as follows. Indoor aspirating was conducted in all bedrooms, kitchens, and other living quarters, while outdoor aspirating was done around structures and near stored debris. Mosquito sampling occurred between 7:00 and 10:00 AM in the morning and 4:00 to 6:30 pm in the evening.

### Field Sites – Texas

Mosquitoes were collected from eight low-income communities called ‘colonias’ in the Lower Rio Grande Valley, South Texas (Fig. 1), as previously described [82]. Between June, 2021 and March, 2022, mosquito sampling was done using BG Sentinel 2 traps (Biogents, Germany) baited with BG lures (Biogents, Germany) placed around homes.

### Mosquito processing, DNA extraction, molecular barcoding

Mosquitoes were morphologically identified to species and sex using illustrations and dichotomous keys [83–85]. Blood-fed females were placed in individual nuclease-free 1.5 mL microcentrifuge tubes and stored at −20 or −80°C with labels indicating species, collection date, and house identification number. These blood-fed mosquitoes were later photographed and assigned a Sella score to record bloodmeal digestion stage and ovary development [86]. To minimize exogenous DNA contamination, each whole mosquito was washed in 10% bleach followed by two rinses with nuclease-free water.

On a new microscope slide (VWR VistaVision microscope slides; VWR, Radnor, PA, USA), the abdomen was carefully separated from the rest of the mosquito (thorax and head) using forceps, and the abdominal contents were transferred into a new labeled, DNA-free 1.5 mL microcentrifuge tube. Forceps were sterilized between specimens. DNA was extracted from gut contents using the Thermo Scientific^™^ KingFisher^™^ Flex Purification System and MagMAX^™^ Core Nucleic Acid Purification Kit (Thermo Fisher Scientific, Waltham, MA, USA), following previously published protocols [24, 87, 88]. DNA from each sample was divided into two aliquots in microcentrifuge tubes to support: (i) molecular verification of mosquito species to confirm morphological identifications, and (ii) bloodmeal metabarcoding.

### Molecular verification of mosquito species

Although field-collected mosquitoes were morphologically identified under a microscope, molecular confirmation was conducted for all blood-fed specimens processed for bloodmeal analysis using a modified cytochrome c oxidase subunit I (COI) barcoding assay using a modified version of the Folmer *et al*. [89] protocol. Briefly, we followed our adapted previously published PCR–Sanger workflow by Oslon et al. (24 ,63,64), the COI region (~ 658 bp) was amplified with primers LCO1490 and HCO2198. PCR reactions (25 μL) contained 12.5 μL FailSafe^™^ PCR 2X Premix E (Lucigen), 0.5 μL FailSafe^™^ PCR Enzyme Mix (Lucigen), 1 μL of each primer, 3 μL DNA template, and 7 μL nuclease-free water.

Thermocycling included an initial denaturation step at 94°C for 3 min, followed by 44 cycles of 94°C for 30 s, 50°C for 30 s, and 72°C for 30 s, and a final extension at 72°C for 8 min. PCR products were purified using Exo-SAP-IT^™^ (Thermo Fisher Scientific) and sequenced bidirectionally at Eton Bioscience (San Diego, CA, USA). Consensus sequences were assembled in Geneious Prime v2024.0 and queried against the NCBI nucleotide database with BLASTn [92]. A match of ≥ 98% identity over ≥ 580 bp was accepted as confirmation. Molecular confirmation was successful for most specimens, and all successfully sequenced specimens matched their original morphological assignments.

### Metabarcoding PCR and sequence analysis

Bloodmeal host identification was performed using a vertebrate 12S rRNA mitochondrial gene following previously published protocols developed and optimized in our laboratory [43, 93]. Briefly, primers with dual identical barcode tags were used to amplify a ~ 145 bp fragment of the mitochondrial 12S rRNA gene [94–96]. Samples were amplified in duplicate. PCR products were pooled, purified using SPRI beads (Beckman Coulter, Indianapolis, IN and Sigma-Aldrich, St. Louis, MO) and submitted to the Texas A&M Institute for Genome Sciences and Society for library preparation (xGen^™^ ssDNA & Low Input DNA Library Prep Kit, Integrated DNA Technologies). Sequencing of samples from Guatemala were done on an Illumina Nextseq 2000 platform and sequencing of the samples from Texas was done on an Illumina Novaseq 6000 platform (Illumina, San Diego, CA, USA).

Sequencing data were processed following our published workflow [43, 93]. Samples were demultiplexed on barcodes using Cutadapt 5.0 [97], primers were trimmed, and reads were merged and quality-filtered using Qiime2 Amplicon 2025.4 [98]. Resulting amplicon sequence variants that had fewer than 100 reads across the study were not considered further. Taxonomic assignment of host sequences was performed with BLAST searches against the NCBI GenBank nucleotide database. Hosts that matched less than 1% of reads for individual samples were also rejected. Only hosts appearing in both PCR replicates were retained, unless one of the replicates did not yield data after processing steps, in which case the available information from one replicate was accepted. Sequences with identity ≥ 98% to database matches were binned to species level. Lower identity matches or multiple species showing identical match values were resolved to the genus or higher taxonomic level. Presence of a host within a geographic region was verified using GBIF (https://www.gbif.org/ Global Biodiversity Information Facility).

### Host quantification and selection analysis

Observed host use for all bloodmeals with single species and mixed (2 or more species) was quantified using relative read abundance (RRA) following Deagle et al [99]. For each mosquito *j* and host species *i*, let *Nij* be the observed read count for host *i* in mosquito *j*. The RRA for host *i* in mosquito *j* was calculated as:

$${RRA}_{ij}=\frac{N_{ij}}{\sum_{k}N_{kj}}$$

where *k* indexes all host species detected in mosquito *j*, and *N_kj_* is the read count for host species *k* in mosquito *j*, so that host proportions sum to 1.0 per mosquito. Mosquitoes were classified as single-host when only one host species was detected; otherwise they were classified as mixed, with meals apportioned fractionally by RRA.

Household-level host availability was obtained from the contemporaneous household census data collected using a questionnaire while visiting homes in Guatemala and Texas (humans, chickens, dogs, ducks, cats, other) [80, 100]. For household *h*, the availability proportion for host *i* was

$$a_{i,h}=\frac{{\text{count}}_{i,h}}{\sum_{k}{\text{count}}_{k,h}}$$

where *count_i,h_* is the number of host *i* recorded in household *h*, and the denominator sums counts across all host categories *k* recorded for that household. Each mosquito was matched to the census of its collection household. Host selection was assessed using mosquito-specific, household-matched forage ratios (FR)[90, 101–103].

$${FR}_{ij}=\frac{{RRA}_{ij}}{a_{i,\text{house}(j)}}$$

where *house* (*j*) denotes the household in which mosquito *j* was collected (i.e., *a*_*i*,*house*(*j*)_ is the same availability proportion defined above for that household). *FR*_*ij*_ was computed only when *a*_*i*,*house*(*j*)_ > 0; if a host category was unrecorded or had zero availability in the household census, *FR* was not estimable for that host. Forage ratios were interpreted as *FR* > 1 indicating over-utilization relative to availability, *FR* < 1 indicating under-utilization, and *FR* ≈ 1 indicating use proportional to availability. For each host, we calculated the mean FR across mosquitoes and reported 95% confidence intervals; evidence of over-/under-utilization was inferred when the 95% CI lay entirely above or below 1, respectively. If the 95% CI included 1, host use was interpreted as not distinguishable from proportional use relative to availability.

For the Texas study site, some property owners allowed trapping but declined the household questionnaire; therefore, FRs were computed only for specimens with matched census, while we report the bloodmeal presence and percentages used for all individual mosquitoes. Maps were generated in R (v4.5.1) using freely available administrative boundaries from Natural Earth, U.S. Census TIGER/Line, and GADM [104–107].

Mixed feeding patterns were compared between mosquito species in Guatemala using Pearson’s chi-square tests with Yates’ continuity correction, specifically testing whether the frequency of mixed feeding differed between *Ae. aegypti* and *Cx. quinquefasciatus* in Guatemala. Statistical significance was assessed at α = 0.05. Analyses were performed in R (v4.5.1)

## Figures and Tables

**Figure 2:**
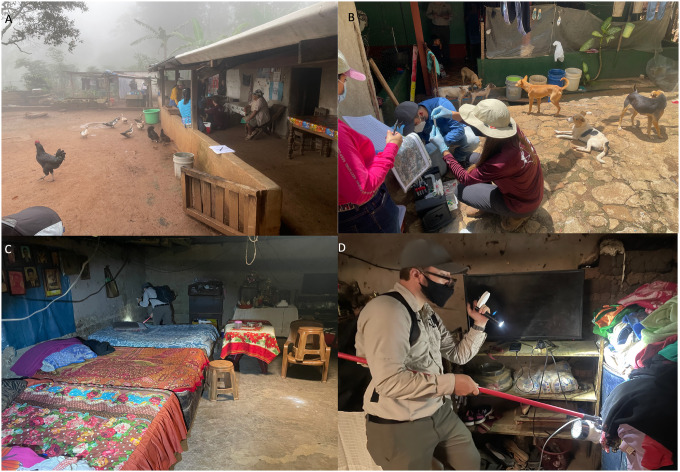
Household and community environments in Comapa, Guatemala. Representative photographs of typical household structures, peridomestic spaces, and community settings in Comapa, Guatemala, where mosquito collections and household surveys were conducted. (A) Typical community/peridomestic setting with open-front home structures and free-ranging domestic animals (e.g., chickens and dogs). (B) Field team conducting household surveys with residents in outdoor living spaces, illustrating common domestic animal presence near human activity. (C) Interior of a typical household showing sleeping areas with minimal barriers to mosquito entry. (D) Indoor mosquito collection using aspiration and a flashlight in a household interior with limited screening.

**Figure 3:**
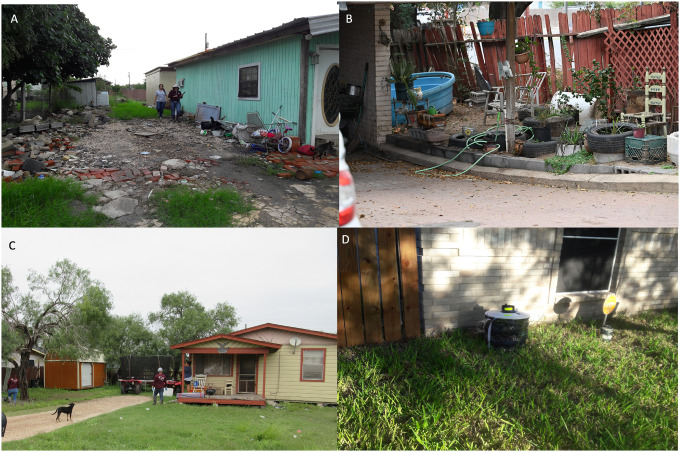
Household and neighborhood environments in South Texas, USA. Representative photographs of residential and peridomestic environments in South Texas, USA, including typical housing and surrounding yard vegetation in neighborhoods where mosquito collections were conducted. (A) Typical residential structure and surrounding yard/peridomestic space in a colonia setting. (B) Backyard environment showing a fenced yard with accumulated items/containers and vegetation that may serve as potential container habitat and mosquito resting sites; domestic dog present. (C) Typical peridomestic yard with a free-roaming domestic dog and surrounding vegetation. (D) BG-Sentinel 2 trap deployed outdoors adjacent to a residence.

**Table 1:** Bloodmeal host composition by mosquito species and collection site.

Hosts detected in mosquito abdomens	*Ae. aegypti* Texas (n = 68)	*Ae. aegypti* Guatemala (n = 41)	*Cx quinquefasciatus* Guatemala (n = 136)
	No. samples (%)	No, samples (%)	No. samples (%)
**Single host species detected:**			
Human (*Homo sapiens)*	2 (2.9%)	29(70.7%)	31(22.8%)
Chicken *(Gallus gallus)*	1(1.5%)	4(9.8%)	15(11.0%)
Dog *(Canis lupus familiaris)*	3(4.4%)	—	—
Turkey *(Meleagris gallopavo)*	—		1(0.7%)
Bird *(*Order *Passeriformes)*	—	—	1(0.7%)
Brown rat *(Rattus norvegicus)*	1(1.5%)	—	—
House mouse *(Mus musculus)*	3(4.4%)	—	—
**Total single host bloodmeal**	**10(14.7%)**	**33(80.5%)**	**48(35.3%)**
**Multiple host species detected:**			
Human + Chicken	—	6(14.6%)	61(44.9%)
Human + Dog	25(36.8)	—	5(3.7%)
Human + Turkey	—	—	1(0.7%)
Human + Bird (*Turdus sp*)	—	2(4.9%)	
Chicken + turkey	—	—	1(0.7%)
Human + Chicken + Dog	—	—	16(11.8)
Human + Chicken + Turkey	—	—	1(0.7%)
Human + Chicken + Bird (*Turdus sp*)	—	—	1(0.7%)
Human + Dog + Cat	17(25%)	—	—
Human + Dog + Rat (*Rattus norvegicus)*	1(1.5%)	—	—
Human + Dog + Cat + Rat	4(5.9%)	—	—
Chicken + Dog	2 (2.9%)	—	2(1.5%)
Dog + Cat (*Felis catus*)	7(10.3)	—	—
Chicken + House mouse	1(1.5%)	—	—
Chicken + Dog + house mouse	1(1.5%)	—	—
**Total multiple host bloodmeal**	**58(85.3%)**	**8 (19.5%)**	**88 (64.7%)**

**Table 2:** Host Selection Patterns: Forage Ratios by Mosquito Species and Location.

Host	Guatemala Ae. aegypti FR (95% CI)	Guatemala Cx. quinquefasciatus FR (95% CI)	Texas *Ae. aegypti* FR (95% CI)
Human	3.62 ± 0.45 (2.70–4.54) *	2.60 ± 0.19 (2.24–2.97) *	0.53 ± 0.14 (0.25–0.81)
Chicken	1.25 ± 0.34 (0.41–2.08)	1.27 ± 0.10 (1.07–1.46)	—
Dog	—	0.99 ± 0.22 (0.51–1.47)	4.65 ± 1.02 (2.43–6.87) *
Cat	—	—	1.55 ± 0.47 (0.52–2.57)

FR = forage ratio (mean ± SE), calculated as the ratio of observed feeding frequency to expected feeding based on host availability from household census data. 95% CI = 95% confidence interval.

Asterisk (*) indicates significant over- or under-utilization (95% CI does not include 1.0).

“—” indicates FR was not estimable due to zero household availability.

## Data Availability

Data from next generation sequencing have been deposited into the SRA database (NCBI) under the accession numbers (Guatemala data: BioProject PRJNA1398928, BioSamples SAMN54467175 to SAMN54467543; Reviewer Link: [https://dataview.ncbi.nlm.nih.gov/object/PRJNA1398928?reviewer=hmoapjnp7tdehaunl8m79t8sm4](https:/urldefense.com/v3/__https:/dataview.ncbi.nlm.nih.gov/object/PRJNA1398928?reviewer=hmoapjnp7tdehaunl8m79t8sm4__;!!KwNVnqRv!Bp12o7tBGXamHOHBaxDVK_VEAHxLx5zoJRlsg7_0y2uTdajBH5mTQ0_10ONsfPavqeROgYzUYVsF9S0$). South Texas data: BioProject PRJNA1417760, BioSamples SAMN55013641 to SAMN55013752; Reviewer Link:[https://dataview.ncbi.nlm.nih.gov/object/PRJNA1417760?reviewer=es3h605apd4dajl26oh7vvtggs] (https:/urldefense.com/v3/__https:/dataview.ncbi.nlm.nih.gov/object/PRJNA1417760?reviewer=es3h605apd4dajl26oh7vvtggs__;!!KwNVnqRv!Bp12o7tBGXamHOHBaxDVK_VEAHxLx5zoJRlsg7_0y2uTdajBH5mTQ0_10ONsfPavqeROgYzUYVsF9jp
